# The Seed of Goal-Related Doubts: A Longitudinal Investigation of the Roles of Failure and Expectation of Success Among Police Trainee Applicants

**DOI:** 10.3389/fpsyg.2019.02151

**Published:** 2019-09-20

**Authors:** Martin Bettschart, Marcel Herrmann, Benjamin M. Wolf, Veronika Brandstätter

**Affiliations:** ^1^Chair for Motivation, Volition, and Emotion, Department of Psychology, University of Zurich, Zurich, Switzerland; ^2^Diagnostics and Counselling, Psychological Institute, School of Applied Psychology, Zurich University of Applied Sciences, Zurich, Switzerland

**Keywords:** personal goals, goal-related doubts, failure, expectation of success, goal striving

## Abstract

Various theories on personal goal striving rely on the assumption that failure raises doubts about the goal. Yet, empirical evidence for an association between objective failure experiences and doubts about personal long-term goals is still missing. In a longitudinal field study, applicants for a job as a police trainee (*n* = 172, *M*_age_ = 25.15; 55 females and 117 males) were accompanied across three measurement times over a period of five months. We investigated the effects of failure and initial expectation of success (in the standardized selection process) on doubts regarding the superordinate goal of becoming a police officer. As hypothesized, both failure and low initial expectation of success as well as their interaction led to increased goal-related doubts over time. The findings provide first empirical evidence for the role of failure in the emergence of goal-related doubts in personal long-term goals and, therefore, the disengagement process as it is hypothesized in various theories on goal striving and life-span development.

## Introduction

Anything seems possible. Time and time again, there are stories in the media about the rise from rags to riches or sensational successes of athletes or businessmen. Such stories make one believe that any goal is attainable if one tries hard enough. However, even exceptionally successful individuals like Michael J. Jordan or Roger Federer know from personal experience that goals are not always attainable.

The present research focuses on the process of becoming aware that a goal may not be achievable (e.g., after repeated failure). Imagine a young man who, as a little boy, had decided to become a police officer. Now, years later, he has applied to several police departments and been rejected every time. In such a case, it may seem adaptive to give up an aspired goal (e.g., Wrosch et al., 2003). However, disengagement from goals is not easy, especially if the goal has been pursued for a long time and considerable effort has been expended. It is argued that goal disengagement is not a single act but “a rather involved process” (Klinger, 1975, p. 8). Research has shown that, between committed goal pursuit and disengagement, individuals go through a phase of doubts and goal re-evaluation (Carver and Scheier, 2005; Brandstätter and Schüler, 2013). This study aims to demonstrate for the first time that failure in the pursuit of a personal long-term goal is an important predictor of goal-related doubts. Furthermore, there is initial evidence indicating that expectation of success is another predictor of doubts (Ghassemi et al., 2017). We try to replicate this finding and additionally investigate a possible interaction effect between failure and expectation of success on doubts.

### Failure as a Predictor of Goal-Related Doubts

Goal-related doubts, on a most fundamental level, are defined as the questioning of the further pursuit of a goal. For example, after the rejection of his application, the young man described above might start to ask himself if he should further pursue the goal of becoming a police officer. Several theories on goal striving postulate such an effect of failure or rather setbacks on doubts and subsequent goal re-evaluation. For example, Klinger (1975) proposes that “unforeseen difficulties” (p. 8) should instigate a process that ultimately leads to goal disengagement. Martin and Tesser (1989) introduced the concept of rumination, which is defined as “conscious thought that results from the disruption of goals” (p. 311). They state that failure in goal pursuit initiates a thought process which “involves attempts to find alternate means to reach important unattained goals or reconciling oneself to not reaching those goals” (p. 311). Similarly, Carver and Scheier (1990) argue that impediments in goal striving may lead to a re-evaluation of the respective goal. Individuals would then “step outside the behavioral stream momentarily and assess the likelihood that the desired outcome will occur, given further effort” (Carver and Scheier, 1990, p. 20). Lastly, Brandstätter and Schüler’s (2013) concept of an action crisis describes a phase of decisional conflict between further goal pursuit and disengagement. An action crisis is thought to emerge when individuals “encounter recurring difficulties” (Brandstätter et al., 2013, p. 1677) in the pursuit of a goal. Despite this large body of theory, failure as a determinant of goal-related doubts has neither been empirically tested (a) with respect to personal long-term goals nor (b) in longitudinal field studies. The present study was designed to close this empirical gap by testing whether failure during personal goal striving leads to increased doubts (Hypothesis 1).

In the course of goal striving, the occurrence of doubts may be predicted not only by objective failure but also by the subjective expectation of success (i.e., goal attainability). Low expectation of success implies the need to re-evaluate a goal in order to determine whether further effort is justified (e.g., Carver and Scheier, 2005). First evidence comes from a longitudinal study on the predictive validity of expectation of success for the multi-faceted experience of an action crisis, part of which are goal-related doubts (Ghassemi et al., 2017). In an attempt to replicate this finding, we hypothesized that low initial expectation of success would lead to increased doubts (Hypothesis 2).

Additionally, failure and expectation of success may have an interactive effect on doubts. Failure should be more likely to result in goal-related doubts if expectation of success is low and setbacks have already been anticipated. In this case, failure confirms the low expectation of success, which should lead to more “certain expectations of continued failure” (Olson et al., 1996, p. 215). This, in turn, provides the ground for doubts and re-evaluation. Hence, we assumed that expectation of success would moderate the effect of failure on doubts, such that doubts are highest when expectation of success had already been low when a failure experience occurred (Hypothesis 3).

### Methodological Challenges in the Study of Personal Long-Term Goals

First indications of the significance of failure for the occurrence of doubts stem from experimental laboratory research using failure feedback on short-term cognitive or motor *tasks* (e.g., Feather, 1966; Beckmann, 1994; Koole et al., 1999). However, it is unclear whether (and if, to what extent) these results can be applied to personal goal striving in everyday life. The affective and cognitive consequences are assumed to be more profound if failure is experienced in self-imposed personal long-term goals, in the pursuit of which time and energy has been invested, compared to failure feedback in experimentally induced tasks. Furthermore, only *quasi-experimental* field studies allow for research on doubts in personal long-term goals as experimentally inducing doubts with respect to these goals might profoundly influence participants’ lives and would therefore not be in line with ethical research standards.

### The Present Research

**FIGURE 1:**
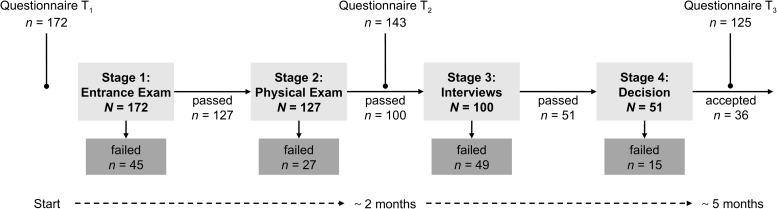
Overview of stages, questionnaires, and sample sizes.

In a longitudinal field study, failure regarding personal goals can be investigated with methodological rigor (i.e., high internal validity) if the goal and the form of failure are identical (i.e., objective/no self-report) for all participants. We conducted a study with participants applying as trainees at a Swiss police department (for a conceptual overview, see the Supplementary Material). Participants did not merely apply for the same type of job at the same institution but pursued the same career goal (i.e., becoming a police officer). The personnel selection process included four stages consisting of standardized tests to evaluate applicants (Figure 1). If applicants failed at a stage, they were allowed to apply at other departments or at the same department again (after a waiting period of 6 months). Failing in the selection process of a police department is seen as a setback in the pursuit of the goal of becoming a police officer and therefore should lead to increased goal-related doubts (Hypothesis 1). Moreover, low initial expectation of success should also lead to increased doubts (Hypothesis 2) and may, additionally, amplify the effect of failure on doubts (Hypothesis 3).

Notably, in some cases, failure may lead to goal disengagement, especially for applicants who have already applied multiple times and/or been experiencing severe goal-related doubts. However, a goal of high personal value typically renders disengagement a lengthy process. Thus, we did not expect a substantial number of unsuccessful applicants to disengage from their goal immediately or shortly after they experienced failure.

## Materials and Methods

### Participants and Procedure

The present sample consisted of applicants from four consecutive and identical selection waves at a Swiss police department. Sixteen participants were excluded because they voluntarily withdrew from the selection process and 10 participants for various other reasons.^1^ The final sample (*n* = 172) comprised 55 female and 117 male applicants (*M*_age_ = 25.15, *SD*_age_ = 3.75, age range = 20–35). This study was carried out in accordance with the ethical guidelines of the APA. The protocol was approved by the Ethics Committee of the Faculty of Arts and Social Sciences of the University of Zurich. All subjects gave written informed consent.

#### Applicant Selection Process

The selection process was divided in four stages (see Figure 1 for an overview of stages, questionnaires, and sample sizes). Before the first stage, applicants had to fill out an online application form and an online self-test to ensure that basic job requirements (e.g., Swiss citizenship, age between 20 and 35 years) were met. If this was the case, applicants were invited to the first stage, the *entrance examination*.

##### Stage 1 – Entrance examination

In the first stage, applicants were required to do various tests and fill out electronic questionnaires at the test center of the police department. The tests and questionnaires were based on established measures and were designed and evaluated by a recruitment consulting firm. Cognitive abilities were tested with an inventory comparable to the Berlin Intelligence Structure Test (Jäger et al., 1997). Additionally, language skills were assessed with three tests (orthography test, grammar test, and dictation). Questionnaires covered integrity (developed based on existing integrity tests; for examples, see Marcus, 2000) as well as several other aspects (e.g., Big Five); however, only integrity was included in the evaluation of stage 1. For each of the three central aspects (cognitive abilities, language skills, and integrity), there were specific cut-off values an applicant had to reach in order to pass this stage.

##### Stage 2 – Physical ability examination

The second stage included five exercises testing different physical abilities (i.e., speed, upper body strength, coordination, trunk muscle strength, and aerobic/anaerobic endurance). For each exercise, there was a maximum of six points. Requirements to gain a certain amount of points for an exercise differed for male and female applicants because an identical examination would have favored male applicants (Birzer and Craig, 1996; Prenzler, 1997). The examination was passed if a minimum of 15 points in total and at least 1 point per exercise was earned.

##### Stage 3 – Interviews

In the third stage, two interviews were conducted, each with one interviewer and two observers of the police department. In both interviews, applicants were asked questions regarding their attitudes and skills (resilience, career choice and motivation, social competence, and taking over responsibility) and their answers were rated by the two observers. Additionally, each observer made a final binary recommendation regarding job suitability based on these ratings. After all interviews of each day, the notes on all applicants were assessed again and a decision was made based on expert consensus.

##### Stage 4 – Final decision round

In the last stage, all applicants that were successful at all previous stages – and thus fulfilled all the prerequisites of the police department to become a police trainee – were ranked by experts in terms of their overall performance in the different stages. Due to capacity limitations, only about two-thirds of the remaining applicants got a job offer. Thus, about one-third of the remaining applicants was rejected at this stage.

#### Study Procedure

Along with the invitation to stage 1, applicants were invited by the police department to participate in the present study in exchange for a compensation of 50 Swiss Francs.^2^ The study was carried out online at three measurement times. The first measurement (T_1_) took place before stage 1, the second measurement (T_2_) between stage 2 and stage 3 (∼2 months after T_1_), and the third measurement (T_3_) after stage 4 (∼5 months after T_1_). T_2_ and T_3_ took place at the same time for those who were still in the process and for those who had dropped out at a stage of the selection process.

As a condition of participation in the study, applicants had to permit access to their objective performance data. This data included the scores of stages 1–3 as well as the information if they passed or failed at each stage and the application as a whole.

### Subjective Psychological Measures

#### Goal-Related Doubts

Goal-related doubts were measured at T_1_–T_3_ with two adapted items of the Action Crisis Scale (Brandstätter and Schüler, 2013). These items focus on two aspects of doubts, namely questioning further goal pursuit (“I doubt whether I should continue striving for my goal of becoming a police officer or disengage from it.”) and having disengagement thoughts (“In the last two weeks, I have thought of disengaging from this goal.”). The other items of the scale were excluded because they capture aspects of hindered goal pursuit different from doubts (e.g., procrastination). The items were assessed on a scale ranging from 1 (*does not apply at all*) to 7 (*applies completely*) and showed a moderate to large correlation (T_1_: ρ = 0.46, T_2_: ρ = 0.64, T_3_: ρ = 0.64; all *p* < 0.001).^3^

#### Expectation of Success

Expectation of success in the selection process was measured at T_1_ with two items on scales ranging from 1 to 7 [e.g., “How likely is it that you pass all the tests of the selection process?” (*very unlikely* to *very likely*)]. The two items correlated moderately (ρ = 0.47, *p* < 0.001).

#### Control Variables

Age, gender, previous applications (PA) at police departments, success importance (Imp), and general self-efficacy (GSE) were used as control variables (all measured at T_1_).

##### Previous applications

A dichotomous variable (0 = no previous applications, 1 = at least one previous application at a police department) was formed to capture if applicants had already applied at a police department in the past. It was used as a control variable to preclude that previous applications had an influence on the effect of failure and expectation of success. However, we did not assume an effect of previous applications as we expected only those with rather high success expectations to apply repeatedly.

##### Success importance

Success importance was used as a control variable because doubts should be less likely to occur for goals with high value, that is, high importance for an individual. Success importance was measured with two items (e.g., “It is important to me to successfully pass the selection process.”) on a scale ranging from 1 (*no agreement*) to 7 (*very much agreement*). The two items correlated moderately (ρ = 0.41, *p* < 0.001).

##### General self-efficacy

General self-efficacy, conceptualized as a relatively stable individual difference, was used as a control variable as it has been shown to be associated with both goal-related doubts (Wolf et al., 2018) and expectation of success (Yeo and Neal, 2006). GSE was measured at T_1_ with two scales of the Inventory on Competence and Control Beliefs (ICCB; Krampen, 1991): self-concept of abilities (e.g., “In order to solve a problem, I can always think of many options.”) and internality (e.g., “When I make a plan, I am sure that it will become reality.”), of eight items each. Only these two scales were used to create a GSE scale as the other two scales do not measure self-efficacy (Shelton, 1990; Chen et al., 2001). The 16 items of the GSE scale were assessed on a scale ranging from 1 (*no agreement*) to 6 (*complete agreement*) and aggregated to a final score of GSE. The scale had a satisfactory internal consistency (α = 0.76).

### Application Measures

The outcome (success or failure) of each of the four stages was used, thus separating five groups of applicants: those who failed at (a) stage 1, (b) stage 2, (c) stage 3, (d) stage 4, and those who (e) succeeded at all stages and received a job offer. In order to operationalize failure, we created four variables with Helmert contrast coding (e.g., Wendorf, 2004). As the five groups were of unequal size, the contrast codes were weighted to ensure orthogonality (Cohen et al., 2003). Table 1 depicts the four variables and the respective values of the groups.

**TABLE 1:** Weighted contrasts for the five groups of applicants failing at different stages of the selection process.

	**Code variables**			
**Group (*n*)**	**FailS1**	**FailS2**	**FailS3**	**FailS4**
Failure at stage 1 (*n*_1_ = 45)	$\frac{\left(n_{2}+n_{3}+n_{4}+n_{5}\right)}{n_{1}}$	0	0	0
Failure at stage 2 (*n*_2_ = 27)	−1	$\frac{\left(n_{3}+n_{4}+n_{5}\right)}{n_{2}}$	0	0
Failure at stage 3 (*n*_3_ = 49)	−1	−1	$\frac{\left(n_{4}+n_{5}\right)}{n_{3}}$	0
Failure at stage 4 (*n*_4_ = 15)	−1	−1	−1	$\frac{n_{5}}{n_{4}}$
Success at all stages (*n*_5_ = 36)	−1	−1	−1	−1

*FailS1 = contrast between applicants who failed vs. succeeded at stage 1. FailS2 = contrast between applicants who failed vs. succeeded at stage 2. FailS3 = contrast between applicants who failed vs. succeeded at stage 3. FailS4 = contrast between applicants who failed vs. succeeded at stage 4.*

## Results

Descriptive statistics of the major study variables are provided in Table 2 (for information on attrition and goal disengagement, see the Supplementary Material).

**TABLE 2:** Means (*SD*s) and zero-order correlations among the major study variables.

**No**	**Variable**	***M* (*SD*)**	**1**	**2**	**3**	**4**	**5**	**6**
1.	Goal-related doubts (T_1_)	1.40 (0.77)	–					
2.	Goal-related doubts (T_2_)	1.44 (0.86)	0.46^∗∗∗^	–				
3.	Goal-related doubts (T_3_)	1.97 (1.33)	0.30^∗∗^	0.43^∗∗∗^	–			
4.	Expectation of success	4.76 (0.98)	–0.12	–0.32^∗∗∗^	–0.12	–		
5.	Success importance	6.51 (0.67)	–0.20^∗∗^	–0.09	–0.04	–0.01	–	
6.	Age	25.15 (3.75)	0.07	0.03	0.08	0.09	–0.03	–
7.	General self-efficacy	72.17 (6.32)	−0.18^∗^	−0.22^∗^	–0.11	0.34^∗∗∗^	0.03	0.10

**N* = 172. T in T_1_−T_*i*_ = measurement time. Doubts regarding the goal of becoming a police officer were only assessed for participants who were still pursuing the goal at T_2_ (*n* = 139) and T_3_ (*n* = 103). ^∗^*p* < 0.05, ^∗∗^*p* < 0.01, ^∗∗∗^*p* < 0.001.*

To analyze the changes in goal-related doubts from T_1_ to T_2_ and from T_2_ to T_3_ in one model, we performed hierarchical linear modeling using R (R Core Team, 2018) and the lme4 package (Bates et al., 2015). First, we entered measurement times, control variables (Age_i_, Gender_i_, PA_i_, Imp_i_, GSE_i_), and doubts at the previous measurement time (to model regression toward the mean; Doubts_it__–__1_) into a random intercept model predicting change in goal-related doubts (ΔDoubts_it_). Second, we added expectation of success (Exp_i_) as well as failure at all four stages (FailS1_i_–FailS4_i_). Third, we added two-way interactions between failure and expectation of success. The full model comprised of the following equations:

Level 1 (measurements *t*):

$$\Delta \,D\,o\,u\,b\,t\,s_{i\,t}=b_{0\,i}+b_{1}\,T\,i\,m\,e_{t}+b_{2}\,D\,o\,u\,b\,t\,s_{i\,t}+\varepsilon_{i\,t}$$

Level 2 (individuals *i*):

$$b_{0\,i}=b_{00}+b_{01}\,A\,g\,e_{i}+b_{02}\,G\,e\,n\,d\,e\,r_{i}+b_{03}\,P\,A_{i}+b_{04}\,I\,m\,p_{i}+b_{05}\,G\,S\,E_{i}+b_{06}\,E\,x\,p_{i}+b_{07}\,F\,a\,i\,l\,S\,1_{i}+b_{08}\,F\,a\,i\,l\,S\,2_{i}+b_{08}\,F\,a\,i\,l\,S\,2_{i}+b_{09}\,F\,a\,i\,l\,S\,3_{i}+b_{10}\,F\,a\,i\,l\,S\,4_{i}+b_{11}\,{\left(F\,a\,i\,l\,S\,1\times E\,x\,p\right)}_{i}+b_{12}\,{\left(F\,a\,i\,l\,S\,2\times E\,x\,p\right)}_{i}+b_{13}\,{\left(F\,a\,i\,l\,S\,3\times E\,x\,p\right)}_{i}+b_{14}\,{\left(F\,a\,i\,l\,S\,4\times E\,x\,p\right)}_{i}+u_{i}$$

Table 3 depicts the results of the full model. Failure at stage 1 [*B* = 0.214, CI_95__%_ (0.099, 0.329)], at stage 2 [*B* = 0.161, CI_95__%_ (0.067, 0.254)], and at stage 3 [*B* = 0.588, CI_95__%_ (0.389, 0.784)] were all significant predictors of an increase in doubts whereas there was no significant effect for failure at stage 4 [*B* = 0.105, CI_95__%_ (−0.073, 0.285)]. Thus, Hypothesis 1 is supported for three of the four stages. Expectation of success was a significant predictor as well [*B* = −0.171, CI_95__%_ (−0.299, −0.044)], supporting Hypothesis 2. Additionally, there was an interaction effect of failure and expectation of success at stage 1 [*B* = −0.135, CI_95__%_ (−0.247, −0.024)] and stage 3 [*B* = −0.282, CI_95__%_ (−0.509, −0.054)], indicating that failure and doubt were more strongly associated when initial expectation of success was low and, thus, partially supporting Hypothesis 3.

**TABLE 3:** Hierarchical linear model predicting the change in goal-related doubts from expectation of success and failure.

	**ΔGoal-related doubts**			
	
			**CI_95%_**	
**Fixed effects**	*****B*****	**(*SE*)**	**Lower**	**Upper**

Intercept	1.385	(1.097)	–0.768	3.562
**Level 1 (within-person)**				
Time	0.484	(0.118)	0.250	0.711
Goal-related doubts (*t*-1)	–0.586	(0.086)	–0.760	–0.414
**Level 2 (between-person)**				
Expectation of success	–0.171	(0.067)	–0.299	–0.044
Fail S1	0.214	(0.059)	0.099	0.329
Fail S2	0.161	(0.047)	0.067	0.254
Fail S3	0.588	(0.102)	0.389	0.784
Fail S4	0.105	(0.092)	–0.073	0.285
Fail S1 × expectation	–0.135	(0.056)	–0.247	–0.024
Fail S3 × expectation	–0.282	(0.115)	–0.509	–0.054

			***CI*_95%_**	
**Random effects**		***SD***	**Lower**	**Upper**

Residual (within-person)		0.866	0.723	0.934
Intercepts (between-person)		0.175	0.000	0.498

**Model fit**		**Coefficient**	***df***	

−2 log likelihood		666.6	17	
AIC		700.6		
BIC		759.9		

**Explained variance**			**Pseudo-$R_{\text{m}}^{2}$**	**Δ$R_{\text{m}}^{2}$**
Model 1: time, control variables, and goal-related doubts (*t*-1)			0.145	0.145
Model 2a: model 1 and expectation of success			0.162	0.017
Model 2b: model 1 and failure at stages 1–4			0.329	0.184
Model 2c: model 1 and both expectation and failures			0.339	0.194
Model 3: model 2c and all failure × expectation interactions			0.368	0.029

**B* = unstandardized regression coefficient; 95% CI = confidence interval for unstandardized regression coefficient based on 9,996 bias-corrected and accelerated bootstrap samples (4 of 10,000 bootstrap runs failed to converge); AIC = Akaike information criterion; BIC = Bayesian information criterion; pseudo-$R_{m}^{2}$ = marginal overall pseudo-*R*^2^ for generalized mixed-effect models (Nakagawa and Schielzeth, 2013); Δ$R_{m}^{2}$ = change in pseudo-*R*^2^ from previous model (i.e., model 1 for models 2a, 2b, and 2c, and model 2c for model 3). Expectation of success was *z*-standardized prior to analysis. Control variables included age, gender, previous applications, success importance, and general self-efficacy. The analysis includes 139 individuals (as 29 individuals only completed T_1_ and 4 individuals reported to have disengaged from their goal at T_2_) and 242 observations. As the interactions between expectation of success and Fail S2 [*B* = −0.055, CI_*95*%_ (−0.144, 0.033)] and Fail S4 [*B* = 0.145, CI_*95*%_ (−0.069, 0.358)] were not significant, the model is reported without them (Hayes, 2013).*

## Discussion

Failure predicted increased goal-related doubts at all stages of the selection process where applicants had an active role (Hypothesis 1). There was no effect for stage 4, which was the final stage of the selection process. In this stage, all previously successful applicants were evaluated in terms of their overall performance in the three preceding stages. Applicants had no active role in this stage, which may have changed the perception of failure. Additionally, in comparison with the preceding stages, this stage was less standardized. It incorporated a non-standardized expert evaluation process of the previous stages resulting in a ranking list of the applicants regarding their suitability for the job. Thus, the informative value of failure for further goal striving might have been lower compared to previous stages.

The evidence for Hypothesis 1 lends empirical support to existing theories on goal striving (e.g., Klinger, 1975; Martin and Tesser, 1989; Carver and Scheier, 1990; Brandstätter and Schüler, 2013). These theories assume that when encountering setbacks in goal striving, it is adaptive to experience doubts and re-evaluate the respective goal. Likewise, in life-span theories, it is argued that setbacks or unsatisfactory goal pursuit lead to more self-centered strategies, which often involve goal re-formation or disengagement. Brandtstädter and Renner (1990) and Brandtstädter and Rothermund (2002), for example, state that discrepancies between an actual and an aspired situation (e.g., due to setbacks) may lead to a form of goal re-formation or, ultimately, goal disengagement (flexible goal adjustment). Heckhausen et al. (2010) argue that an accumulation of setbacks would lead to a shift from active (primary) to passive (secondary) control strategies. This shift is assumed to be associated with distress and goal re-formation processes. The present research can also be embedded in other theories of goal setting and goal striving, such as the mindset theory of action phases (Gollwitzer, 2012). This theory distinguishes four consecutive phases concerned with the deliberation about possible goals, the planning and implementation of goal-directed actions, and the evaluation of goal-related outcomes. In the terminology of mindset theory, doubts could be conceptualized as a backward loop resulting again in the deliberation about the goal itself causing a “mindset shift” from an implemental to a deliberative mindset (Brandstätter and Schüler, 2013; Gollwitzer, 2018).

Replicating the findings of Ghassemi et al. (2017), expectation of success at the beginning of the selection process predicted an increase in doubts (Hypothesis 2). This is in line with theories suggesting that expectation of success (or attainability) of goals is a main determinant of effort and persistence (Carver and Scheier, 1990; Jostmann and Koole, 2009; Gendolla et al., 2012). Low expectation should thus make individuals more susceptible to goal-related doubts, instigating re-evaluation in order to discard unworthy goals.

There was some evidence for an interactive effect of failure and expectation of success on the change in doubts in stages 1 and 3 (Hypothesis 3). This indicates that a highly attainable goal may be (at least temporarily) protected from doubts even when difficulties arise. This goal shielding would be adaptive as it would allow for persistent goal striving (Feather, 1989). However, if setbacks accumulate, it might be maladaptive to hold onto an appraisal of high expectation of success and to show unchanged persistence. This unproductive persistence would lead to “the expenditure of time and effort with no more success than would have occurred had one quit early” (Feather, 1989, p. 69). Above that, valued alternatives for goal striving may be ignored (Feather, 1989).

### Limitations

Some limitations of this study have to be discussed. First, there seemed to be selective attrition. Among the 47 participants who did not fill out all the questionnaires, 44 experienced failure in the selection process. Despite encouraging all applicants to complete the study, we had expected that rejected applicants would show a higher attrition rate than successful applicants. Because they had been rejected, willingness to further participate in the study may have been diminished. Furthermore, some of the applicants probably refrained from further participating because they were experiencing severe doubts or had even disengaged from their goal and did not want to deal with potentially unpleasant questions regarding their discontinued career plans. Thus, it is more plausible that the effects were restricted rather than generated by an artifact of attrition.

Second, we cannot rule out the possibility that rejected applicants succeeded at or failed in the selection process of another police department between two measurements. If this was the case, it may have affected their experience of doubts.

## Conclusion

The present study makes an important contribution to research on personal goals as it validates a general self-regulatory mechanism in an applied context. To the best of our knowledge, it was the first longitudinal field study examining the effects of objective failure in a personal long-term goal on goal-relevant variables. Individuals who encountered failure in goal striving experienced more doubts about their goal. However, failure did not result in goal-related doubts if expectation of success had been comparably high, implying that high expectation of success at the outset of an endeavor shields individuals from doubts when setbacks occur. Future studies are needed to further investigate this moderating effect. Furthermore, because of the possible negative impact of goal-related doubts on well-being and performance (e.g., Brandstätter et al., 2013; Herrmann and Brandstätter, 2015), it is crucial to develop interventions that help individuals to prevent or cope with these doubts. To protect an individual from doubts after a setback, expectation of success may be increased via self-training or in coaching sessions. This might be particularly indicated when individuals pursue career goals where setbacks are immanent (e.g., academia, sports) and when objective criteria strongly suggest that a goal is attainable.

## Data Availability Statement

The datasets generated for this study are available on request to the corresponding author.

## Ethics Statement

The research was conducted in accordance with the ethical guidelines of the APA. The protocol was approved by the Ethics Committee of the Faculty of Arts and Social Sciences of the University of Zurich.

## Author Contributions

All authors made significant contributions to the conceptualization and design of this research, contributed to the manuscript revision, and read and approved the submitted version of the manuscript. MB collected the data, performed the statistical analysis, and wrote the manuscript.

## Conflict of Interest

The authors declare that the research was conducted in the absence of any commercial or financial relationships that could be construed as a potential conflict of interest. The reviewer FW declared a shared affiliation, though no other collaboration, with one of the authors, MH, to the handling Editor.

## Footnotes

1 Seven of these 10 participants were excluded because they filled out questionnaire T_1_ after stage 1 or T_2_ after stage 3 contrary to instructions. One participant was excluded because she/he did not appear to have filled out the questionnaires seriously, one participant because of a failed mandatory background check performed by the police department between T_1_ and T_2_, and one participant because of a failed mandatory medicine check before stage 4.

2 We expected that failed applicants would have diminished commitment to the police department and, as a consequence, willingness to further participate at the study. To reduce attrition, applicants were informed at the beginning of the study that they would only receive compensation if they completed all three questionnaires and they were encouraged to complete the study irrespective of success or failure in the selection process.

3 Analyses showed skewed distributions for doubts and success importance. Therefore, Spearman’s correlations are reported.

## References

[B1] Bates D. Mächler M. Bolker B. Walker S. (2015). Fitting linear mixed-effects models using lme4. *J. Stat. Softw.* 67 1–48. 10.18637/jss.v067.i01

[B2] Beckmann J. (1994). Ruminative thought and the deactivation of an intention. *Motiv. Emot.* 18 317–334. 10.1007/BF02856472

[B3] Birzer M. L. Craig D. E. (1996). Gender differences in police physical ability test performance. *Am. J. Police* 15 93–108. 10.1108/07358549610122494

[B4] Brandstätter V. Herrmann M. Schüler J. (2013). The struggle of giving up personal goals: affective, physiological, and cognitive consequences of an action crisis. *Pers. Soc. Psychol. Bull.* 39 1668–1682. 10.1177/0146167213500151 23976776

[B5] Brandstätter V. Schüler J. (2013). Action crisis and cost–benefit thinking: a cognitive analysis of a goal-disengagement phase. *J. Exp. Soc. Psychol.* 49 543–553. 10.1016/j.jesp.2012.10.004

[B6] Brandtstädter J. Renner G. (1990). Tenacious goal pursuit and flexible goal adjustment: explication and age-related analysis of assimilative and accommodative strategies of coping. *Psychol. Aging* 5 58–67. 10.1037/0882-7974.5.1.58 2317302

[B7] Brandtstädter J. Rothermund K. (2002). The life-course dynamics of goal pursuit and goal adjustment: a two-process framework. *Dev. Rev.* 22 117–150. 10.1006/drev.2001.0539

[B8] Carver C. S. Scheier M. F. (1990). Origins and functions of positive and negative affect: a control-process view. *Psychol. Rev.* 97 19–35. 10.1037/0033-295X.97.1.19

[B9] Carver C. S. Scheier M. F. (2005). “Engagement, disengagement, coping, and catastrophe,” in *Handbook of Competence and Motivation*, eds Elliot A. J. Dweck C. S. (New York, NY: Guilford Publications), 527–547.

[B10] Chen G. Gully S. M. Eden D. (2001). Validation of a new general self-efficacy scale. *Organ. Res. Methods* 4 62–83. 10.1177/109442810141004

[B11] Cohen J. Cohen P. West S. G. Aiken L. S. (2003). *Applied Multiple Regression/Correlation Analysis for the Behavioral Sciences*, 3rd Edn Mahwah, NJ: Lawrence Erlbaum Associates Publishers.

[B12] Feather N. T. (1966). Effects of prior success and failure on expectations of success and subsequent performance. *J. Pers. Soc. Psychol.* 3 287–298. 10.1037/h0022965 5906331

[B13] Feather N. T. (1989). “Trying and giving up: persistence and lack of persistence in failure situations,” in *Self-defeating Behaviors: Experimental Research, Clinical Impressions, and Practical Implications*, ed. Curtis R. C. (New York, NY: Plenum Press), 67–95. 10.1007/978-1-4613-0783-9_4

[B14] Gendolla G. H. E. Wright R. A. Richter M. (2012). “Effort intensity: some insights from the cardiovascular system,” in *The Oxford Handbook of Human Motivation* ed. Ryan R. M. (New York, NY: Oxford University Press), 420–438.

[B15] Ghassemi M. Bernecker K. Herrmann M. Brandstätter V. (2017). The process of disengagement from personal goals. *Pers. Soc. Psychol. Bull.* 43 524–537. 10.1177/0146167216689052 28903660

[B16] Gollwitzer P. M. (2012). “Mindset theory of action phases,” in *Handbook of Theories of Social Psychology*, eds van Lange P. A. M. Kruglanski A. W. Higgins E. T. (Thousand Oaks, CA: Sage Publications), 526–545. 10.4135/9781446249215.n26

[B17] Gollwitzer P. M. (2018). The goal concept: a helpful tool for theory development and testing in motivation science. *Motiv. Sci.* 4 185–205. 10.1037/mot0000115

[B18] Hayes A. F. (2013). *Introduction to Mediation, Moderation, and Conditional Process Analysis: A Regression-Based Approach.* New York, NY: Guilford Press.

[B19] Heckhausen J. Wrosch C. Schulz R. (2010). A motivational theory of life-span development. *Psychol. Rev.* 117 32–60. 10.1037/a0017668 20063963 PMC2820305

[B20] Herrmann M. Brandstätter V. (2015). Action crises and goal disengagement: longitudinal evidence on the predictive validity of a motivational phase in goal striving. *Motiv. Sci.* 1 121–136. 10.1037/mot0000016

[B21] Jäger A. O. Süss H.-M. Beauducel A. (1997). *Berliner Intelligenzstruktur-Test. BIS-Test, Form 4 [Berlin Test of Intelligence Structure, BIS-Test, Form 4].* Göttingen: Hogrefe.

[B22] Jostmann N. B. Koole S. L. (2009). “When persistence is futile: a functional analysis of action orientation and goal disengagement,” in *The Psychology of Goals*, eds Moskowitz G. B. Grant H. (New York, NY: Guilford Press), 337–361.

[B23] Klinger E. (1975). Consequences of commitment to and disengagement from incentives. *Psychol. Rev.* 82 1–25. 10.1037/h0076171

[B24] Koole S. L. Smeets K. van Knippenberg A. Dijksterhuis A. (1999). The cessation of rumination through self-affirmation. *J. Pers. Soc. Psychol.* 77 111–125. 10.1037/0022-3514.77.1.111

[B25] Krampen G. (1991). *Fragebogen zu Kompetenz- und Kontrollüberzeugungen [Inventory for the Measurement of Self-Efficacy and Externality].* Göttingen: Hogrefe.

[B26] Marcus B. (2000). *Kontraproduktives Verhalten im Betrieb: Eine individuumsbezogene Perspektive [Counterproductive behavior in organizations: An individual-based perspective].* Göttingen: Hogrefe.

[B27] Martin L. L. Tesser A. (1989). “Toward a motivational and structural theory of ruminative thought,” in *Unintended Thought*, eds Uleman J. S. Bargh J. A. (New York, NY: Guilford Press), 306–326.

[B28] Nakagawa S. Schielzeth H. (2013). A general and simple method for obtaining R2 from generalized linear mixed-effects models. *Methods Ecol. Evol.* 4 133–142. 10.1111/j.2041-210x.2012.00261.x 30239975

[B29] Olson J. M. Roese N. J. Zanna M. P. (1996). “Expectancies,” in *Social Psychology: Handbook of Basic Principles*, eds Higgins E. T. Kruglanski A. W. (New York, NY: Guilford Press), 211–238.

[B30] Prenzler T. (1997). Problem oriented approach to preventing sex discrimination in police recruitment. *Crime Prev. Stud.* 7 221–237.

[B31] R Core Team (2018). *R: A Language and Environment for Statistical Computing.* Vienna: R Foundation for Statistical Computing.

[B32] Shelton S. H. (1990). Developing the construct of general self-efficacy. *Psychol. Rep.* 66 987–994. 10.1177/003329419006600301

[B33] Wendorf C. A. (2004). Primer on multiple regression coding: common forms and the additional case of repeated contrasts. *Underst. Stat.* 3 47–57. 10.1207/s15328031us0301_3

[B34] Wolf B. M. Herrmann M. Brandstätter V. (2018). Self-efficacy vs. action orientation: comparing and contrasting two determinants of goal setting and goal striving. *J. Res. Pers.* 73 35–45. 10.1016/j.jrp.2017.11.001

[B35] Wrosch C. Scheier M. F. Carver C. S. Schulz R. (2003). The importance of goal disengagement in adaptive self-regulation: when giving up is beneficial. *Self Identity* 2 1–20. 10.1080/15298860309021

[B36] Yeo G. B. Neal A. (2006). An examination of the dynamic relationship between self-efficacy and performance across levels of analysis and levels of specificity. *J. Appl. Psychol.* 91 1088–1101. 10.1037/0021-9010.91.5.1088 16953770

