# Supplementary material for: The Seed of Goal-Related Doubts: A Longitudinal Investigation of the Roles of Failure and Expectation of Success Among Police Trainee Applicants
Source: Front Psychol. 2019 Sep 20;10:2151. doi: 10.3389/fpsyg.2019.02151 (PMC6764325; doi:10.3389/fpsyg.2019.02151)
Supplement: Supplementary file 1 [file Data_Sheet_1.PDF]

Supplemental material to Bettschart, Herrmann, Wolf, & Brandstätter

The Seed of Goal-Related Doubts: A Longitudinal Investigation of the Roles of Failure and Expectation of Success Among Police Trainee Applicants

Conceptual Overview of the Study

Figure S1 depicts a conceptual overview of the study including the main variables.

Attrition and Goal Disengagement

To investigate systematic attrition, applicants who filled out all questionnaires (n = 125) vs. applicants who did not fill out all questionnaires (n = 47; attrition group) were compared regarding the main study variables. Applicants of the attrition group failed significantly more often at stage 1, $\chi^2(1) = 37.37$, $p < .001$, and in the selection process in general, $\chi^2(1) = 8.27$, $p = .004$. Among the n = 47 applicants of the attrition group, n = 44 experienced failure in the selection process. Additionally, applicants of the attrition group (M = 5.01, SD = 1.13) showed higher expectation of success than applicants who filled out all the questionnaires (M = 4.66, SD = 0.90), $t(170) = 2.11$, $p = .036$. There were no differences between the two groups regarding the other study variables (all $p > .18$).

Applicants who failed at a stage were asked if they still pursued their goal. Table S1 depicts the number of applicants who indicated to have disengaged or not disengaged from their goal at T_2 and T_3. In total, n = 84 rejected applicants filled out the questionnaire at T_3; of these, n = 16 applicants (19%) indicated goal disengagement.

Table S1

Number of Rejected Applicants Indicating Disengagement From Their Goal to Become a Police Officer at T_2 and T_3

	Disengagement at T_2		Disengagement at T_3	
Stage of failure	no	yes	no	yes
Stage 1 or 2	42	4	27	6[a]
Stage 3 or 4	-	-	41	10
All stages	-	-	68	16

Note. Applicants were only asked if they disengaged from their goal if they had been rejected at a stage.
[a] Three of these applicants already disengaged at T_2.

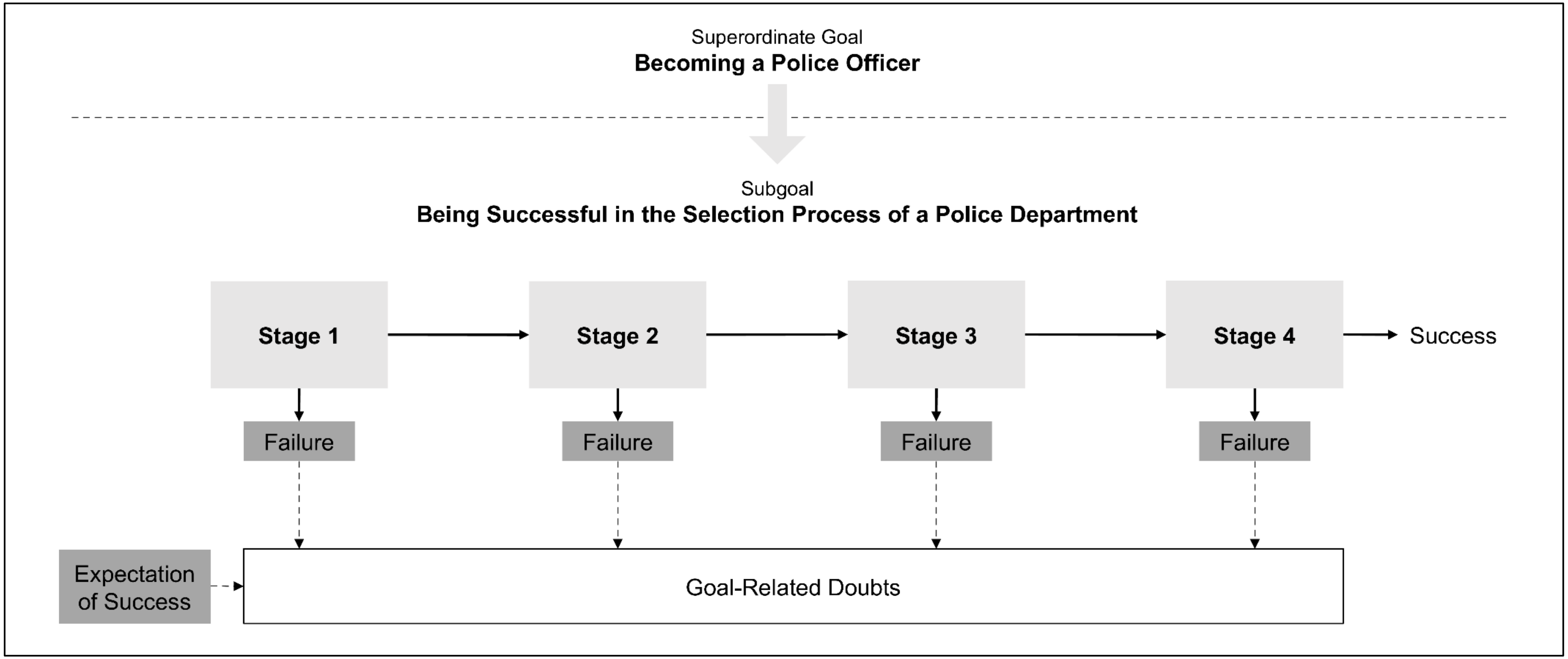

Figure S1. Conceptual overview of the study.
